# Editorial: Responses and adaptation of plants to abiotic stress: genetics, evolution and molecular insights

**DOI:** 10.3389/fgene.2025.1729869

**Published:** 2025-11-24

**Authors:** Yang Tao, Xilan Yang, Zhiqiang Wang

**Affiliations:** 1 Sichuan-Xizang Medicinal Resource Breeding and Standardization Team, Institute for Advanced Study, Chengdu University, Chengdu, China; 2 Coarse Cereal Research Institute, Chengdu Agricultural College, Chengdu, China; 3 VIB Center for Plant Systems Biology, Ghent University, Ghent, Belgium

**Keywords:** abiotic stress adaptation, crop improvement, multi-omics integration, genome editing and gene stacking, genetics

Climate change is intensifying abiotic stresses like drought, heat, and salinity, which severely threaten crop productivity and global food security (Raza et al., 2025; Zandalinas et al., 2021). Breeding climate-resilient crops has therefore become an urgent imperative. This Research Topic was organized to highlight advances in understanding how plants respond and adapt to harsh environments at genetic, evolutionary, and molecular levels, and to bridge fundamental discoveries with crop improvement.

Several contributions in this Research Topic focus on discovering genes and pathways that confer stress tolerance. Xiong et al. characterized a cell wall-loosening gene, *ZmEXPB7*, from maize. Overexpressing *ZmEXPB7* promoted deeper rooting, reduced water loss (via lower stomatal density and faster closure), and improved survival under water deficit in *Arabidopsis*, and thus enhanced drought tolerance. This finding underscores the crucial role of expansin proteins in stress adaptation and positions *ZmEXPB7* as a promising target for engineering drought-resilient crops. On a genome-wide scale, Yuan et al. systematically identified 105 kinesin (*KIN*) motor protein gene family in pea (*Pisum sativum*). Expression profiling revealed that several members (e.g., *PsKIN8*, *PsKIN54*) are strongly upregulated under drought, while others (*PsKIN47*, *PsKIN51*) respond to salt stress. These stress-responsive kinesins are potential candidates for improving plant stress tolerance. Evolutionary analysis further revealing segmental duplications and conserved motifs in the KIN family, which suggests these motor proteins have been maintained by selection due to their importance in stress adaptation.

Another study expanded our view to the whole-plant and transcriptome level by examining a naturally stress-resistant species. Wei et al. applied an integrative long- and short-read transcriptomics approach to the desert shrub *Nitraria tangutorum*, unveiling its transcriptional and physiological responses to drought. They revealed an early and coordinated activation of stress signaling pathways: notably plant hormone (abscisic acid) signaling and MAPK cascades. Metabolic adjustments, including enhanced starch–sucrose interconversion and elevated amino acid and lignin biosynthesis, also emerged as key adaptive strategies in this xerophyte. Several transcription factor families (AP2/ERF, WRKY, bHLH, NAC, MYB) were prominently involved in regulating drought-responsive genes. Comprehensive physiological measurements (e.g., proline, H_2_O_2_, malondialdehyde levels, and antioxidant enzyme activities) further illuminated how *N. tangutorum* withstands severe dehydration. Importantly, network analysis identified hub genes that likely act as master regulators of drought tolerance (through hormone signaling, ROS scavenging, and other protective processes). These findings enlarge the genomic resources for an extremophile plant and provide novel molecular targets that could be transferred into crops to boost their drought resilience.

Complementing the biological insights, one contribution presented a data-driven phenotyping innovation with direct implications for crop breeding. Liu et al. developed a method combining hyperspectral image and machine learning to accurately identify yellow-seeded *Brassica napus* (rapeseed), a variety desirable for its lower anti-nutritional compounds and higher oil and protein content. Their logistic regression model reached approximately 98% classification accuracy for yellow versus non-yellow seeds, matching human experts and previous approaches. Support vector machine and random forest classifiers performed similarly, each exceeding 95% accuracy. This study illustrates how advanced sensing and AI techniques can streamline the selection of important traits. By enabling rapid, non-destructive screening of seeds, such tools can accelerate breeding programs–a concept that could be extended to detect stress-tolerant phenotypes or other quality traits in crops.

Taken together, the research in this Topic spans from single-gene studies to systems-level analyses and emerging technologies, all aiming to enhance plant stress resilience. A unifying thread is crop improvement: insights into expansins, motor proteins, and regulatory networks can inform crop engineering and marker-assisted selection, while advances in phenotyping and genomics support smarter breeding decisions.

Looking ahead, key priorities include understanding how plants respond to combined stresses, such as drought and heat, which often occur simultaneously in the field. Integrated *pan-omics* approaches—encompassing genomics, transcriptomics, proteomics, and metabolomics—will be essential for unraveling the complexity of stress responses (Mansoor and Chung, 2024; Raza et al., 2025). Translating lab findings into field-ready solutions is equally crucial, requiring validation under real-world, multi-stress conditions (Ganie and Azevedo, 2025). Next-generation breeding technologies will drive further gains. Artificial intelligence and machine learning can mine large datasets to uncover gene–trait relationships, accelerating the development of stress-tolerant varieties (Crossa et al., 2025). Genome editing enables gene stacking for broad-spectrum resilience (Bailey-Serres et al., 2019; Li et al., 2025), while genetic resources from wild relatives and extremophiles offer untapped potential for enhancing hardiness (Farooq et al., 2025). Finally, synthetic biology, including programmable gene circuits, may allow intelligent reprogramming of stress responses in crops (Borowsky and Bailey-Serres, 2024).

In conclusion, this Research Topic highlights a forward-looking trajectory for plant stress biology. Continued efforts along these interdisciplinary lines–from gene discovery and evolutionary insight to field validation and high-tech breeding–will drive the development of climate-resilient, high-yielding crops. The work assembled here both deepens our understanding of plant responses to abiotic stress and provides new insights for sustaining agricultural productivity in a changing world.
